# Arbitrary absolute vs. individualized running speed thresholds in team sports: A scoping review with evidence gap map

**DOI:** 10.5114/biolsport.2023.122480

**Published:** 2023-02-01

**Authors:** Filipe Manuel Clemente, Rodrigo Ramirez-Campillo, Marco Beato, Jason Moran, Adam Kawczynski, Piotr Makar, Hugo Sarmento, José Afonso

**Affiliations:** 1 Escola Superior Desporto e Lazer, Instituto Politécnico de Viana do Castelo, Rua Escola Industrial e Comercial de Nun’Álvares, 4900-347 Viana do Castelo, Portugal; 2 Instituto de Telecomunicações, Delegação da Covilhã, Lisboa 1049-001, Portugal; 3 Exercise and Rehabilitation Sciences Institute. School of Physical Therapy. Faculty of Rehabilitation Sciences. Universidad Andres Bello. Santiago, Chile; 4 School of Health and Sports Sciences, University of Suffolk, Ipswich, UK; 5 School of Sport, Rehabilitation and Exercise Sciences, University of Essex, Colchester, Essex, UK; 6 Gdańsk University of Physical Education and Sport, Poland; 7 University of Coimbra, Research Unit for Sport and Physical Activity (CIDAF), Faculty of Sport Sciences and Physical Education, Coimbra, Portugal; 8 Centre of Research, Education, Innovation, and Intervention in Sport (CIFI2D), Faculty of Sport, University of Porto, Portugal

**Keywords:** Sports, Athletic performance, Geographic information systems, Soccer, Football, Exercise test, Human physical conditioning

## Abstract

The aims of this scoping review were (i) to characterize the main methodological approaches to assessing individualized running speed thresholds in team sports players; (ii) to assess the use of traditional arbitrary (absolute) thresholds compared to individualized running speed thresholds in team sports players; (iii) to provide an evidence gap map (EGM) about the approaches and study designs employed in investigations in team sports and (iv) to provide directions for future research and practical applications for the strength and conditioning field. Methods studies were searched for in the following databases: (i) PubMed; (ii) Scopus; (iii) SPORTDiscus and (iv) Web of Science. The search was conducted on 15/07/2022. Risk of bias was assessed using the Risk of Bias Assessment Tool for Nonrandomized Studies (RoBANS). From 3,195 potentially relevant articles, 36 were eligible for inclusion in this review. Of the 36 included articles, 27 (75%) focused on the use of arbitrary and individualized running speed thresholds to describe the locomotor demands (e.g., high intensity running) of players. Thirty-four articles used individualized speed running thresholds based on physical fitness assessments (e.g., 40-m linear sprint) or physical performance (e.g., maximal acceleration). This scoping review supported the need for a greater focus to be placed on improving the methodological aspects of using individualized speed running thresholds in team sports. More than just creating alternatives to arbitrary thresholds, it is essential to increase the replicability of methodological conditions whilst ensuring that research comparing the most adequate measures and approaches to individualization takes into consideration the population and context of each study.

## INTRODUCTION

Monitoring the locomotor demands of team sport players during training sessions and matches is a common practice by coaches and has become a popular research topic over the last decade [1–3]. The evolution of microelectromechanical systems such as Global Positioning Systems (GPS), Local Position Systems, Ultrawide Band or inertial measurement units have facilitated accurate evaluations of the locomotor demands that are placed on team sport players [4–6]. This evolution has enabled coaches and researchers alike to characterize both the volume and intensity that players must sustain during training and competition [7, 8]. The importance of monitoring these demands has been recognized by coaches, sports scientists and players as observed in a survey, conducted in soccer, in which all stakeholders reported GPS-derived training data as being at least ‘somewhat important’ [9]. In another survey of strength and conditioning coaches working in professional soccer, it was reported that 94% of the respondents reported using GPS-based technologies for the above mentioned purposes in their sport [10].

A concern regarding the monitoring of locomotor demands in sport is the establishment of appropriate running speed thresholds that facilitate the proper quantification of intensity and volume [11]. Despite variability in fitness levels, a recurrent practice in the monitoring of locomotor demands is the use of arbitrary (player-independent) running speed thresholds [12, 13]. The use of arbitrary thresholds is often necessitated by software-based constraints that typically require fixed running speeds for analytical purposes. However, such thresholds are also required due to the methodological challenges associated with the individualization of running speeds which may vary from player to player and from sport to sport. Whilst arbitrary absolute running speed thresholds may allow coaches to benchmark players’ values (across different contexts) and simplify the data monitoring process, they may also impede the individualization of training prescription because running speed can be physical fitness and context-dependent [14]. Moreover, although using arbitrary running speed thresholds has become common practice, these thresholds are not consistent across measurement instruments and contexts thus making it very difficult to summarize evidence in this domain [15].

The use of individualized running speed thresholds has been proposed as a way of overcoming the weaknesses associated with arbitrary thresholds [16]. This process is based on the physical fitness level of the individual player, aiming to mitigate between-player variability through the identification of a unique running speed threshold [17]. However, the selection of appropriate methods of individualization based on physical fitness levels represents a primary challenge that has been observed in the literature [18]. Different approaches have been utilized with individualized thresholds being based on maximal aerobic speed (MAS) [11], maximal sprint speed (MSS) [19] and anaerobic speed reserve (ASR) [14]. Besides the diversity of approaches used to establish individualized thresholds, other challenges have also emerged with, for example, the calculation of MAS being dependent on the type of test used to determine an athlete’s performance [20]. On this, different tests used for establishing MAS (e.g., laboratory, field-based) have failed to find consistent agreement across analyses using gold-standard methods [21, 22]. Moreover, the specificity of the test (e.g., distance-based or time-based) can affect the derived MAS value [20] and, still more, since ASR is MAS-dependent [20], the ASR method is similarly compromised. Using MSS can also be challenging due to minimal changes in performance occurring over the course of a season [23] and because sprinting threshold is affected by the biomechanical profile of the individual [13].

An alternative approach to the development of physical-fitness-based thresholds is the use of a data analytics approach to defining running speed zones [24]. As an example, a study conducted in female soccer players used a *k*-mean, Gaussian mixture model and spectral clustering to define four running speed zones based on information extracted from players over 52 international soccer matches. In another example, spectral clustering was used to determine velocity thresholds in Gaelic football referees [25].

The diversity of approaches for defining running speed thresholds in team sport players is apparent in the extant literature [12, 26, 27]. Identifying how these methodological approaches have been established and tracking the technologies as they develop may help researchers and practitioners to define the next steps in standardizing thresholds within and between sports. To date, no scoping review has been conducted on the body of literature relating to individualized running speed thresholds in team sport players. Such a review is necessary in terms of mapping the extant literature and facilitating an evaluation of the landscape of the methodological approaches. Accordingly, the purposes of this study are to: (i) characterize the main methodological approaches to assessing individualized running speed thresholds in team sports players; (ii) assess the use of traditional arbitrary (absolute) thresholds compared to individualized speed thresholds in team sports players; (iii) provide an evidence gap map (EGM) on the approaches and study designs adopted in team sports; and (iv) provide future directions for research and practical applications for the sports science field.

## MATERIALS AND METHODS

This scoping review followed the PRISMA 2020 guidelines [28] and took into consideration the recommendations for scoping reviews checklist (PRISMA-ScR) [29].

### Protocol and registration

The scoping review protocol was preliminarily submitted and published on the Open Science Framework, with the registration number 10.17605/OSF.IO/92HWU, on 15^th^ July 2022. The protocol can be accessed via the web address https://osf.io/bt5nc/?view_only=42c3045fb7ba42ef97fb58f359719f6c, as well as through the registration number 10.17605/OSF.IO/92HWU.

### Eligibility criteria

Studies published in peer-reviewed journals, including those with the status of “in press” or “ahead-of-print”, were considered. No date limitations were set, and studies undertaken in all languages were considered [30]. The eligibility criteria were established based on the PECOS (population, exposure, comparator, outcome, study design) approach: (i) population: team sports players, of any age, male or female, who were integrated into team training routines (i.e., not injured or with any reported pathology or health problems). Excluded were disabled athletes or those competing in adapted sports. (ii) exposure: exposed to analysis of individualized running speed thresholds in training sessions and/or matches; (iii) comparator: exposed to traditional arbitrary running speed thresholds in training sessions and/or matches; (iv) outcome(s): the time and/or distance and/or percentage of time and/or distance spent in different running speed thresholds (either in arbitrary/absolute or individualized thresholds); (v) study design: observational studies or interventions (both single-arm [if with two different metrics, for example, individualized vs. arbitrary] and multi-arm investigations were considered).

### Information sources

The following databases were searched: PubMed, Scopus, SPORT-Discus and Web of Science (Core collection). After performing the protocol registration (ID: 10.17605/OSF.IO/92HWU), the searches were conducted on the same day (15/07/2022). In addition to the database searches, manual searches were performed on the reference lists of included studies to identify potentially relevant titles. The abstracts of these articles were checked for relevant inclusion criteria and, if necessary, the full-text was referred to. Snowballing citation tracking, preferentially in Web of Science, was also conducted whilst two external experts (as recognized by Expertscape at the *Worldwide* level: https://expertscape.com/ex/soccer) were also consulted. Finally, errata and article retractions were analyzed for any articles that were included in the review [31].

### Search strategy

In the search, the Boolean operators AND/OR were applied. No filters or limitations were used (e.g., date; language; study design) to maximize the chances of identification of appropriate studies [32]. The main search strategy was as follows:

[Title/Abstract] bandy OR baseball* OR basketball* OR cricket OR floorball* OR football* OR futsal OR handball* OR hockey OR hurling OR korfball* OR lacrosse OR netball* OR polo* OR rugby OR “sepak takraw” OR soccer OR softball* OR slamball* OR “Team Sport*” OR volleyball*

AND

[All fields/Full text] individual* OR personalized

AND

[All fields/Full text] speed OR velocity OR quickness OR intensity OR running OR sprint*

AND

[All fields/Full text] threshold* OR zone*

The full search strategy can be observed in the following Table 1.

**TABLE 1:** Full search strategy for each database.

Database	Specificities of the databases	Search Strategy
PubMed	None to report	(((bandy[Title/Abstract] OR baseball*[Title/Abstract] OR basketball*[Title/Abstract] OR cricket[Title/Abstract] OR floorball*[Title/Abstract] OR football*[Title/Abstract] OR futsal[Title/Abstract] OR handball*[Title/Abstract] OR hockey[Title/Abstract] OR hurling[Title/Abstract] OR korfball*[Title/Abstract] OR lacrosse[Title/Abstract] OR netball*[Title/Abstract] OR polo*[Title/Abstract] OR rugby[Title/Abstract] OR “sepak takraw”[Title/Abstract] OR soccer[Title/Abstract] OR softball*[Title/Abstract] OR slamball*[Title/Abstract] OR “Team Sport*”[Title/Abstract] OR volleyball*[Title/Abstract]) AND (individual* OR personalized)) AND (speed OR velocity OR quickness OR intensity OR running OR sprint*)) AND (threshold* OR zone*)

Scopus	Search for title and abstract also includes keywords	( TITLE-ABS-KEY ( bandy OR baseball* OR basketball* OR cricket OR floorball* OR football* OR futsal OR handball* OR hockey OR hurling OR korfball* OR lacrosse OR netball* OR polo* OR rugby OR “sepak takraw” OR soccer OR softball* OR slamball* OR “Team Sport*” OR volleyball* ) AND ALL ( individual* OR personalized ) AND ALL ( speed OR velocity OR quickness OR intensity OR running OR sprint* ) AND ALL ( threshold* OR zone* ) )

SPORTDiscus	None to report	AB ( bandy OR baseball* OR basketball* OR cricket OR floorball* OR football* OR futsal OR handball* OR hockey OR hurling OR korfball* OR lacrosse OR netball* OR polo* OR rugby OR “sepak takraw” OR soccer OR softball* OR slamball* OR “Team Sport*” OR volleyball* ) AND TX ( “individual*” OR personalized ) AND TX ( speed OR velocity OR quickness OR intensity OR running OR sprint* ) AND TX ( threshold* OR zone* )

Web of Science	Search for title and abstract also includes keywords and its designated “topic”	

### Selection process

Two of the authors (HS and JA) independently screened the retrieved records (namely titles and abstracts). The same authors also independently screened the gathered full texts. Disagreements between the two authors were discussed in a joint reanalysis. In the case of no consensus being reached, a third author (FMC) made the final decision. Where and when required, all co-authors shared opinions with regard to any doubts raised in the selection process, with a view to supporting the final decision. The EndNote^TM^ 20.3 software (Clarivate^TM^) was used for managing records, namely the removal of duplicates either automatically or manually.

### Data extraction process

The data extraction process was firstly performed by the lead author (FMC) and was verified by two co-authors (RRC and HS) to confirm the accuracy and details of the data. A specially designed Microsoft^®^ Excel datasheet was created and used to contain the data and the main information. The Excel datasheet can be observed in the supplementary material. In the case of relevant data being missing from a full text of a study, the primary author (FMC) directly contacted the corresponding author of that study by email and/or ResearchGate to obtain the required information.

### Data items

The descriptive characteristics of participants that were collected were sport, age, sex, competitive level and training frequency. The characterization of competitive level followed the Participant Classification Framework [33]: Tier 0: sedentary (not included in our context); Tier 1: recreationally active (not included in our context); Tier 2: trained/developmental; Tier 3: highly trained/national level; Tier 4: elite/international level; Tier 5: world class.

Context-related information: this included, but was not restricted to, period of the season, context of the assessment (period of rest before analysis, time of the day), the number of sessions/matches considered.

Methodological-related included the method used for the individualization (e.g., MAS, ASR, MSS) and the arbitrary/absolute running speed thresholds that were collected. It also included information about the instruments of measurement such as GPS, local positioning systems, or ultrawide band, and the regularity of the tests performed (if more than once).

Main outcome: considering the goal of executing a scoping review with an EGM, the main outcomes were those associated with the methodological approaches of the studies and not the specific results presented in each article. Accordingly, running speed thresholds were the variable of interest.

Additional information: this included, but was not limited to, citation details, year, country of data collection, funding sources, and competing interests.

### Study risk of bias assessment

The risk of bias was independently assessed by two authors (JA and HS). In the case of disagreements, both reanalyzed the process. In the case of no subsequent consensus being reached, a third author (FMC) made the final decision. The Risk of Bias Assessment Tool for Nonrandomized Studies (RoBANS) was used to assess the risk of bias of the included studies [34]. This scale has shown moderate reliability and good feasibility and validity [34]. The tool comprises six domains: the selection of participants; confounding variables; the measurement of exposure; the blinding of the outcome assessments; incomplete outcome data; and selective outcome reporting. The domains are classified as ‘low’, ‘high’ and ‘unclear’ risk of bias [34] (Table 2).

**TABLE 2:** Risk of bias assessment of non-randomized studies.

Study	Selection of participants	Confounding variables	Measurement of exposure	Blinding of outcome assessments	Incomplete outcome data	Selective outcome reporting
Abbott et al. [51]	Low	Unclear	Unclear	Low	Low	Low
Abt et al. [26]	Low	Unclear	Unclear	Low	Low	Low
Beato et al. [57]	Low	Unclear	Unclear	Low	Low	Low
Carling et al. [75]	Low	Unclear	High	Low	Low	Low
Casamichana et al. [76]	Low	Unclear	Low	Low	Low	Low
Castellano et al. [77]	Low	Unclear	Unclear	Low	Low	Low
Castellano et al. [78]	Low	Unclear	Unclear	Low	Low	Low
Clarke et al. [38]	Low	Unclear	Unclear	Low	Low	Low
Darbellay et al. [43]	Low	Unclear	High	Low	Low	Low
Fitzpatrick et al. [11]	Low	Unclear	Low	Low	Low	Low
Gabbett et al. [45]	Low	Unclear	High	Low	Low	Low
Gamble et al. [79]	Low	Unclear	Low	Low	Low	Low
Goto et al. [80]	Low	Unclear	High	Low	Low	Low
Hunter et al. [19]	Low	Unclear	Unclear	Low	Low	Low
Jastrzebski et al. [39]	Low	Unclear	Low	Low	Low	Low
Jastrzebski et al. [40]	Low	Unclear	Low	Low	Low	Low
Lovell et al. [41]	Low	Unclear	Unclear	Low	Low	Low
Martínez-Cabrera et al. [49]	Low	Unclear	Unclear	Low	Low	Low
Martínez-Cabrera et al. [50]	Low	Unclear	Unclear	Low	Low	Low
Massard et al. [81]	Low	Unclear	High	Low	Low	Low
Murray et al. [44]	Low	Unclear	High	Low	Low	Low
Nakamura et al. [52]	Low	Unclear	Low	Low	Low	Low
Núñez-Sánchez et al. [82]	Low	Unclear	Unclear	Low	Low	Low
O’Connor et al. [56]	Low	Unclear	High	Low	Low	Low
Ortega-Gálvez et al. [42]	Low	Unclear	High	Low	Low	Low
Park et al. [24]	Low	Low	Low	Low	Low	Unclear
Rago et al. [46]	Low	Unclear	High	Low	Low	Low
Rago et al. [14]	Low	Unclear	High	Low	Low	Low
Reardon et al. [16]	Low	Unclear	High	Low	Low	Low
Scanlan et al. [83]	Low	Unclear	High	Low	Low	Low
Scott et al. [17]	Low	Unclear	Unclear	Low	Low	Low
Scott et al. [47]	Low	Unclear	Unclear	Low	Low	Low
Siegle et al. [54]	Low	Low	Low	Low	Low	Low
Taylor et al. [48]	Low	Unclear	Unclear	Low	Low	Low
Taylor et al. [84]	Low	Unclear	Unclear	Low	Low	Low
Taylor et al. [85]	Low	Unclear	Low	Low	Low	Low

### Data management and synthesis methods

An EGM was built to graphically represent the type of studies and the evidence collected on the main topic of research. The EGM summarized the findings and provided a brief overview of the evidence and research gap [35–37]. A narrative review also accompanied the results, while specific information about the number and/or percentage of studies and the topics of interest was outlined. Table 1 presents an example how information was collected regarding the scoping review context and outcomes.

## RESULTS

### Study identification and selection

**FIG. 1:**
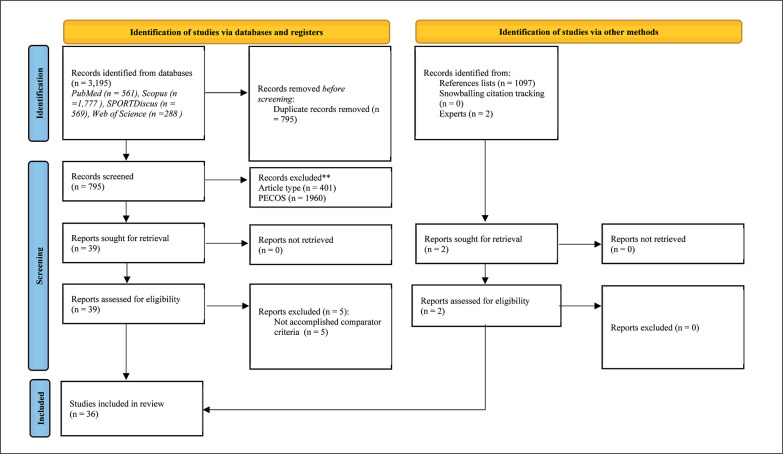
PRISMA 2020 flow diagram.

The initial search yielded a total of 3,195 titles (Figure 1). The data were imported to the EndNote^TM^ reference manager software (version 20.2, Clarivate Analytics, Philadelphia, PA, USA). Duplicates (795 titles) were subsequently removed, either automatically or manually. The remaining 2,400 titles were screened for their relevance based on their titles and abstracts. Of those, 2,361 titles were removed. The full texts of the remaining 39 titles were then inspected and from there, five more were removed based on the reasons presented in Supplementary material #1. After the automatic search, 34 articles remained for data extraction and further analysis. Following revision of the list of 34 articles by the experts, two further eligible titles were suggested, reviewed, and integrated. In total, 36 articles were included in the final scoping review.

### Methodological quality

With regard to risk of bias, confounding variables were unclear in 34 out of 36 (94.4%) of the articles as physical fitness assessments and performance measures were not controlled in relation to recovery status, hours of sleep, diet, nutritional supplementation on the day of assessment or monitoring of well-being. The risk of bias in the measurement of exposure was high in 12 of 36 (33.3%) of the included articles as physical fitness assessments were not conducted in close time proximity (> 4 weeks) to the point of data collection. This may compromise the individualization of running speed thresholds because adaptations can occur in the elapsed time between the test and the collection of data. Moreover, the risk of bias in the measurement of exposure was unclear in 15 of 36 (41.7%) of the included studies because the assessment was not conducted on a regular basis (< 1 week), or the identification of the process was not made clear by the authors. All of the included studies presented a low risk of bias in the selection of participants, and in the blinding of outcome assessments as the blinding process was not considered to exert an influence on the final outcomes in these studies (i.e., experimental studies). In addition, incomplete data was of low risk of bias in the included studies.

### Study characteristics and context-related information

The characteristics and context-related information of the included studies can be observed in Table 3. Seventeen out of 36 studies (47.2%) used only match-related data, while 10 from 36 studies (27.8%) used only training session data. Five out of 36 studies (13.9%) used both training and match-related data. Thirty-one of 36 (86.1%) used GPS systems to collect data and 4 from 36 studies (11.1%) used multicamera tracking systems. Just one study (2.8%) used local positioning systems.

**TABLE 3:** Characterization and contextual information of the included studies.

Study	Sport	Comp. Level	N	Age (years)	Sex	Country	Training frequency (per week)	Training sessions analyzed	Matches analyzed	Period of observation	Regularity of the physical assessment	Instrument used
Abbott et al. [51]	Soccer	Tier 3	31	19.4 ± 1.7	M	UK	4–5	23	4	4 weeks	1 (pre-season)	OptimEye (10 Hz, GPS)

Abt et al. [26]	Soccer	Tier 3	10	27 ± 5	IU	UK	IU	0	3	IU	1 (3^rd^ week of the season)	ProZone (multicamera tracking system)

Beato et al. [57]	Soccer	Tier 4	20	28.4 ± 4.3	M	Italy	IU	IU	6	42 days	1	Apex STATSports (10 Hz, GPS)

Carling et al. [75]	Soccer	Tier 4	12	25 ± 3	IU	France	IU	0	31	One season	1 (beginning of the season)	AMISCO Pro (multicamera tracking system)

Casamichana et al. [76]	Field H.	Tier 4	16	25.5 ± 2.9	M	Spain	4	0	17	Two seasons	Every match	GPSport (10 Hz, GPS)

Castellano et al. [77]	Soccer	Tier 2	24	13.3 ± 0.5	IU	Spain	4	9	0	5 weeks	1 (before observation period)	MinimaxX (10 Hz, GPS)

Castellano et al. [78]	Soccer	Tier 2	44	12.1 ± 0.4 and 13.3 ± 0.5	IU	Spain	3–4	9	0	5 weeks	1 (before observation period)	MinimaxX (10 Hz, GPS)

Clarke et al. [38]	Rugby	Tier 4	12	23.5 ± 4.9	W	Australia	IU	0	6	First round of the season	1	GPSport (5 Hz, interpolated to 15 Hz GPS)

Darbellay et al. [43]	Soccer	Tier 4	88	26.5 ± 5.8	M	UK	IU	IU	IU	First round of the season	1	FieldWiz (10 Hz, GPS)

Fitzpatrick et al. [11]	Soccer	Tier 3	14	17 ± 1	IU	Switzerland	4–5	2	13	6 weeks	2 (start and end of the observation period)	MinimaxX S4 (10 Hz, GPS)

Gabbett et al. [45]	Rugby	Tier 2	90	13.7 ± 0.9	M	Australia	IU	0	18	IU	1	MinimaxX (10 Hz, GPS)

Gamble et al. [79]	Ice H.	Tier 2	46	20.0 ± 1.4 and 21.9 ± 1.1	M; W	Canada	IU	IU	24	One season	Every match	Kinexon (20 Hz, LPS)

Goto et al. [80]	Soccer	Tier 2	81	10.2 to 16.2	IU	UK	3	0	IU	IU	1 (start of he season)	GPSport (1 Hz, GPS)

Hunter et al. [19]	Soccer	Tier 3	12	Under-18	IU	UK	5–6	0	22	Two seasons	4 (6 in 6 weeks)	MinimaxX (5 Hz, GPS)

Jastrzebski et al. [39]	Soccer	Tier 4	16	27.5 ± 4.1 and 19.1 ± 3.1	M; W	Poland	5–7	1	0	2 weeks	1	MinimaxX 4.0 (10 Hz, GPS)

Jastrzebski et al. [40]	Soccer	Tier 3	13	27.1 ± 5.2	IU	Poland	5–7	2	0	2 weeks	1	MinimaxX 4.0 (10 Hz, GPS)

Lovell et al. [41]	Soccer	Tier 3	8	24 ± 5	IU	UK	IU	IU	IU	Two seasons	IU | 6 in 6 weeks	ProZone (multicamera tracking system)

Martínez-Cabrera et al. [49]	Soccer	Tier 3	26	17.3 ± 1.1	IU	Spain	4–5	0	18	IU	1	GPSport Pro X (15 Hz, GPS)

Martínez-Cabrera et al. [50]	Soccer	Tier 3	26	17.3 ± 1.1	IU	Spain	4–5	0	18	IU	1	GPSport Pro X (15 Hz, GPS)

Massard et al. [81]	Soccer	Tier 2	47	22.9 ± 4.1	IU	Australia	3	67	23	One season	2 (end of preseason and mid of the season)	MinimaxX S4 (10 Hz, GPS)

Murray et al. [44]	Aust. Foot.	Tier 4	45	22 ± 3	IU	Australia	IU	IU	IU	39 weeks	1 (beginning of the season)	Optimeye S5 (10 Hz, GPS)

Nakamura et al. [52]	Soccer	Tier 3	11	21.0 ± 3.0	W	Brazil	IU	0	10	IU	1 (beginning of the season)	GPSport SPI Elite (5 Hz, GPS)

Núñez-Sánchez et al. [82]	Soccer	Tier 2	20	26.6 ± 4.1	IU	Spain	14 hours/ week	0	4	Pre-season	1	GPSport SPI Pro (15 Hz, GPS)

O’Connor et al. [56]	Aust. Foot.	Tier 3	53	24.4 ± 3.7	M	Australia	IU	114	0	Two preseasons and one and half in-season	IU	Optimeye S5 (10 Hz, GPS)

Ortega-Gálvez et al. [42]	Field H.	Tier 3	15	23.7 ± 4.1	W	Spain	IU	0	4	Two months	1	GPSport SPI HPU (15 Hz, GPS)

Park et al. [24]	Soccer	Tier 5	27	24.6 ± 3.8	W	USA	IU	0	52	Three years		MinimaxX S4 (10 Hz, GPS)

Rago et al. [46]	Soccer	Tier 3	13	25.8 ± 3.5	M	Italy	IU	42	3	8 weeks	1	BT-Q1000 Ex, QStarz (10 Hz, GPS)

Rago et al. [14]	Soccer	Tier 3	13	25.8 ± 3.5	M	Italy	IU	45	0	8 weeks	1	BT-Q1000 Ex, QStarz (10 Hz, GPS)

Reardon et al. [16]	Rugby	Tier 3	36	27.2 ± 3.9	IU	Ireland	IU	0	20	9 months	1	V5 and S5 Catapult (10 Hz, GPS)

Scanlan et al. [83]	Basketball	Tier 2	13	20.4 ± 4.6	M	Australia	2–4	10–25	0	6 weeks (pre-season)	1	Optimeye S5 (10 Hz, GPS)

Scott et al. [17]	Soccer	Tier 4	22	28.3 ± 21.9–39.5	W	IU	IU	21	0	21 days	1	Optimeye S5 (10 Hz, GPS)

Scott et al. [47]	Soccer	Tier 3	36	23.2 to 25.3	W	USA	IU	0	11.6	Two pre-seasons		Optimeye S5 (10 Hz, GPS)

Siegle et al. [54]	Soccer	Tier 5	IU	Adults	M	Italy France	IU	0	1	1 day	1	ASpoGAMo system (25 Hz, multicamera tracking system)

Taylor et al. [48]	Soccer	Tier 2	31	15.3 to 18.9	IU	UK	3–12	5 weeks	IU	5 weeks	1	Statsports APEX (10 Hz, GPS)

Taylor et al. [84]	Rugby	Tier 3	10	18.4 ± 1.0	IU	UK	4–6	7.8	6	6 weeks	1	Catapult S5 (10 Hz, GPS)

Taylor et al. [85]	Rugby	Tier 3	12	17 to 18	IU	UK	IU	3	0	3 weeks	3	Catapult S5 (10 Hz, GPS)

**FIG. 2:**
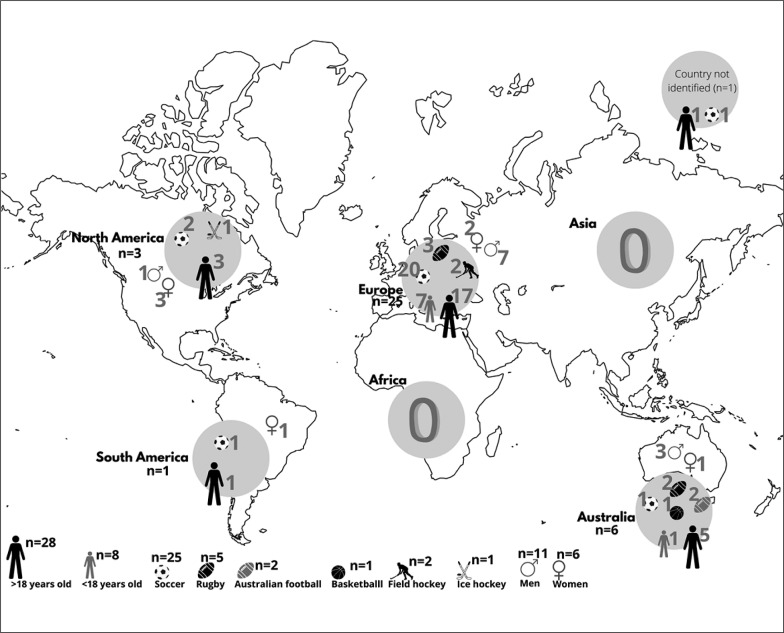
Distribution of the included studies per continent, age-group and team sport.

Aust. Foot.: Australian Football; Comp.: competitive; GPS: Global Positioning System; H: hockey; IU: information unavailable; M: men; N: number of participants; UK: United Kingdom; USA: United States of America; W: women;

Figure 2 presents the distribution of the included studies per continent, age-group and team sport. Of the 36 included articles, 69.4% were conducted in soccer, 13.9% in rugby, 5.6% in Australian football, 5.6% in field hockey, 2.8% in basketball and 2.8% in ice hockey. Twenty-five studies (69.4%) were conducted in European populations while six studies (16.7%) were conducted in Australians. Three studies were conducted in North America (8.3%), and one in South America (2.8%), while one study did not report location information (2.8%). Twenty-eight (77.8%) of the studies were conducted in populations with average ages greater than 18 years old, while eight studies (22.2%) were undertaken in participants below 18 years old. In relation to competitive level, 17 studies (47.2%) were conducted in populations from tier 3, nine studies from tier 2 (25.0%), eight studies from tier 4 (22.2%), and two studies from tier 5 (5.6%). Ten studies (27.8%) were exclusively conducted in men, six studies (16.7%) were exclusively conducted in women and two studies (5.6%) were conducted in both sexes. Eighteen studies (50%) did not report the sex of the participants.

**FIG. 3:**
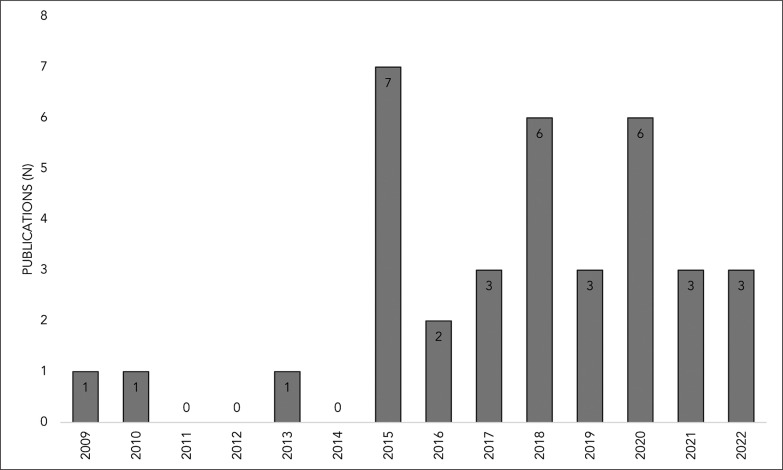
Articles published per year related to individualized running speed thresholds.

Figure 3 presents the included articles published per year relating to individualized running speed thresholds. Of the 36 studies included, 58.3% (n = 21) were published in the last five years (2018–2022), while the year with the most publications was 2015 (n = 7; 19.4%).

### Methodological characteristics of the included studies

Table 4 summarizes the main methodological characteristics of the included studies. Of the 36 included articles, 34 (94.4%) used individualized running speed thresholds based on physical fitness assessments or physical performance while two (5.6%) used alternative approaches (Q-Q-plots for visual inspection of intersection points and machine learning algorithms). Of the included studies, 15 (41.7%) used maximal sprint speed as a measure to individualize the running speed thresholds, while 11 studies (30.6%) used a combination of MAS, MSS, and ASR. Five studies (13.9%) used respiratory compensation threshold or second ventilatory threshold and two (5.6%) used MAS for individualization of running speed thresholds.

**TABLE 4:** Methodological characteristics of the included studies.

Study	Objective of research	Tests used for individualization	Method of individualization	Arbitrary thresholds used	Individualized thresholds used
Abbott et al. [51]	Analyze the differences between arbitrary and individualized acceleration thresholds in training sessions and matches	40-m linear sprint	Maximum rate of acceleration during 40-m linear sprint	Low-intensity acceleration (1–2 m/s^2^); Moderate-intensity acceleration (2–3 m/s^2^); High-intensity acceleration (> 3 m/s^2^).	Low-intensity acceleration (25–50% of maximal acceleration); Moderate-intensity acceleration (50–75% of maximal acceleration); High-intensity acceleration (> 75 of maximal acceleration).

Abt et al. [26]	Compare arbitrary and individualized speed thresholds on locomotor outcomes in matches	Incremental treadmill test	The 2^nd^ ventilatory threshold was used to determine the point of high intensity speed threshold.	High intensity running (distances covered at > 19.8 km/h)	High intensity running (2^nd^ ventilatory threshold)

Beato et al. [57]	Analyze the workload of professional soccer players using arbitrary and individualized outcomes	Peak speed registered in training sessions/ matches	Peak speed attained in training sessions/matches	Distance covered above 20 km/h Distance covered above 25 km/h	High-speed distance (80% MSS)

Carling et al. [75]	Analyze the variability of locomotor demands between matches	Incremental treadmill test	MAS was determined and used to classify match activities	Total high-speed running distance (average running speed ≥ 19.8 km/h); High speed running distance (average running speed from 19.8 to 25.2 km/h); Total sprint distance (average running speed > 25.2 km/h).	Between 80–100% MAS; > 100% MAS; ≥ 80% MAS.

Casamichana et al. [76]	Compare arbitrary and individualized speed thresholds on locomotor outcomes in matches	Peak speed in match	Peak speed attained during the season (in match)	Moderate speed running (15.1–18.9 km/h); High speed running (> 19 km/h); Very high speed running (> 24 km/h); Sprint running (> 30 km/h).	Distances covered at ~30%, 50%, 60%, 75% and 95% of the average peak speed of the players.

Castellano et al. [77]	Analyze the influence of different small-sided games on locomotor and physiological outcomes in small-sided games	30-m linear sprint	Individualized to MSS	Distance at 0–3 km/h; Distance at 3–8 km/h; Distance at 8–13 km/h; Distance at 13–16 km/h; Distance at > 16 km/h.	Distance at > 40% MSS; Distance at 40–60% MSS; Distance at > 60% MSS.

Castellano et al. [78]	Analyze the influence of different small-sided games on locomotor and physiological outcomes in small-sided games	30-m linear sprint	Individualized to MSS	Distance at 0–3 km/h; Distance at 3–8 km/h; Distance at 8–13 km/h; Distance at 13–16 km/h; Distance at > 16 km/h.	Distance at > 40% MSS; Distance at 40–60% MSS; Distance at > 60% MSS.

Clarke et al. [38]	Compare arbitrary and individualized speed thresholds on locomotor outcomes in matches	Incremental treadmill test	The 2^nd^ ventilatory threshold was estimated and used for the individualization of high intensity running.	High intensity running (5 m/s); High intensity running (group mean 2^nd^ ventilatory threshold); Low-speed running (< 2 m/s); Sprint (acceleration at > 2.5 m/s^2^ for a minimum of 1 second).	High intensity running (individualized to the 2^nd^ ventilatory threshold)

Darbellay et al. [43]	Compare arbitrary and individualized speed thresholds on locomotor outcomes in small-sided games and matches	Yo-Yo Intermittent RecoveryTest level 1 and 40-m linear sprint	The MAS was obtained from the latest stage achieved in the progressive running test, while the ASR was estimated based on the difference between MSS and the MAS. Additionally, the metabolic power (resulted from speed and acceleration data) was also used as individualized method.	Low intensity (0 to 8 km/h); Moderate intensity (8 to 13 km/h); Intermediate speed (13 to 16 km/h); High intensity (16 to 19 km/h); Very high intensity (> 19 km/h).	Low intensity (0 to 60% MAS or 0 to 10 W/kg metabolic power zone); Moderate intensity (60 to 80% MAS or 10 to 15 W/kg metabolic power zone); Intermediate speed (80 to 100% MAS or 15 to 20 W/kg metabolic power zone); High intensity (100% MAS to 30% ASR or 20 to 35 W/kg metabolic power zone); Very high intensity (> 30% ASR or > 35 W/kg metabolic power zone).

Fitzpatrick et al. [11]	Compare dose-response relationship between arbitrary and individualized speed threshold with changes in aerobic fitness	1500-metre time trial and 40-m linear sprint	The MAS was obtained from the 1500-metre time trial, while the ASR was estimated based on the difference between MSS and the MAS.	High speed distance (above 17 km/h) - match to group average of MAS; High speed distance (above 21 km/h) - match to group average of 30% ASR.	Meters covered above MAS; Meters covered above 30% ASR.

Gabbett et al. [45]	Compare arbitrary and individualized speed thresholds on locomotor outcomes in matches	40-m linear sprint	The MSS in the interval of 20-40-linear sprint test was obtained to estimate the peak velocity.	Low speed (0-3.5 m/s); Moderate speed (3.6-5.0 m/s); High speed (> 5.0 m/s).	Low speed (0-25% MSS); Moderate speed (25-50% MSS) High speed (50-70% MSS); Very high speed (> 70% of the peak velocity).

Gamble et al. [79]	Compare arbitrary and individualized speed thresholds on locomotor outcomes in matches	Peak speed in match	Peak speed attained during the season (in match)	1.0–10.9 km/h; 11.0–13.9 km/h; 14.0–16.9 km/h; 17.0–20.9 km/h; 21.0–24.0 km/h; > 24 km/h	< 20% of the peak speed; 20-39% of the peak speed; 40–59% of the peak speed; 60–79% of the peak speed; 80–90% of the peak speed; > 90% of the peak speed.

Goto et al. [80]	Compare arbitrary and individualized speed thresholds on locomotor outcomes in matches	10-m linear sprint	The flying 5-m linear sprint time was used to estimate the MSS and to split into five equal individualized speed zones	Speed zone 1 (0.0-0.5 m/s); Speed zone 2 (1.6-3.0 m/s); Speed zone 3 (3.1-4.5 m/s); Speed zone 4 (4.6-6.0 m/s); Speed zone 5 (> 6.0 m/s).	Speed zone 1 (slowest: 0.0–1.1 m/s; fastest 0.0–1.5 m/s); Speed zone 2 (slowest: 1.2–2.2 m/s; fastest 1.6–3.0 m/s); Speed zone 3 (slowest: 2.3–3.3 m/s; fastest 3.1–4.5 m/s); Speed zone 3 (slowest: 3.4–4.4 m/s; fastest 4.6–6.0 m/s); Speed zone 3 (slowest: > 4.4 m/s; fastest > 6.0 m/s).

Hunter et al. [19]	Compare arbitrary and individualized speed thresholds on locomotor outcomes in matches	Incremental treadmill test and 40-m linear sprint	MAS and the RCT were obtained from the incremental treadmill test and were used as measures for individualization. The MSS was obtained in the fastest 10-m split. The ASR resulted from the subtraction of MSS by the MAS.	Low speed running (< 14.99 km/h); High-speed running (15.0-17.99 km/h); Very-high speed running (18.0-24.99 km/h); Sprinting (25.0-35.0 km/h).	Low speed running (< RCT or < 79%MAS; or < 49%MSS; or < 79% MAS); High-speed running (RCT-95% of maximal oxygen uptake; or 80-99% MAS; or 50-59% MSS; or 80-99% MAS); Very-high speed running (95% of maximal oxygen uptake-29% ASR; or 100-139% MAS; or 60-79% MSS; or 100% MAS-29% ASR); Sprinting (30% ASR-MSS; or 140% MAS-35 km/h; or 80-100% MSS; or 30% ASR-MSS)

Jastrzebski et al. [39]	Compare arbitrary and individualized speed thresholds on locomotor outcomes in small-sided games	Incremental treadmill test and 40-m linear sprint	The lactate threshold was estimated during the incremental treadmill test, while applying the Dmax method. The MSS was determined in the 40-m linear sprint.	Standing, walking (0-2 m/s); Jogging (2-4 m/s); Running (4-5.5 m/s); High-speed running (5.5-7 m/s); Sprinting (> 7 m/s).	Walking (0-1 m/s); Walking, jogging (1-2 m/s); Low intensity running (2 m/s-lactate threshold); High-intensity running (lactate threshold-80%MSS); Sprinting (> 80% MSS).

Jastrzebski et al. [40]	Compare arbitrary and individualized speed thresholds on locomotor outcomes in small-sided games	Incremental treadmill test and 40-m linear sprint	The lactate threshold was estimated during the incremental treadmill test, while applying the Dmax method. The MSS was determined in the 40-m linear sprint.	Standing, walking (0-2 m/s); Jogging (2-4 m/s); Running (4-5.5 m/s); High-speed running (5.5-7 m/s); Sprinting (> 7 m/s).	Walking (0-1 m/s); Walking, jogging (1-2 m/s); Low intensity running (2 m/s-lactate threshold); High-intensity running (lactate threshold-80%MSS); Sprinting (> 80% MSS).

Lovell et al. [41]	Compare arbitrary and individualized speed thresholds on locomotor outcomes in match	Incremental treadmill test	The 1^st^ ventilatory threshold, the RCT and MAS were estimated during the incremental treadmill test.	High-speed running (> 14.4 km/h); Very-high speed running (> 19.8 km/h).	Low (< 1^st^ ventilatory threshold); Moderate (1^st^ ventilatory threshold-RCT); High (RCT-MAS).

Martínez-Cabrera et al. [49]	Compare arbitrary and individualized speed thresholds on high-intensity acceleration in match	40-m linear sprint starting from standing, 6, 10.8 and 15 km/h	The maximal acceleration attained in the 40-m linear sprint test was used for the individualization.	High-intensity acceleration (> 3 m/s^2^).	High-intensity acceleration (> 75% maximal acceleration).

Martínez-Cabrera et al. [50]	Compare arbitrary and individualized speed thresholds on high-intensity acceleration in match	40-m linear sprint starting from standing, 6, 10.8 and 15 km/h	The maximal acceleration attained in the 40-m linear sprint test was used for the individualization.	High-intensity acceleration (> 3 m/s^2^); High-intensity acceleration (> 4 m/s^2^).	High-intensity acceleration (> 75% maximal acceleration & > 21 km/h); High-intensity acceleration (> 75% maximal acceleration).

Massard et al. [81]	Compare arbitrary and individualized speed thresholds regarding the impact on workload measures and non-contact injury	40-m linear sprint starting or peak speed attained in match demands (players not assessed for 40-m sprint) and 30-15 Intermittent Fitness test for MAS	The final velocity achieved at 30-15 Intermittent Fitness test was used as reference for the high-speed running individualized threshold.	High-speed running (19.8 km/h or 5.5 m/s^2^).	High-speed running (> MAS)

Murray et al. [44]	Compare arbitrary and individualized speed thresholds regarding the impact on injury likelihood and workload measures	Peak speed attained in training demands	The average MSS (32.1 km/h) was used as reference to create the relative thresholds for each speed zone.	Low (< 6 km/h); Moderate (6-18 km/h); High (18-24 km/h); Very high (> 24 km/h).	Low (0-19.99% MSS); Moderate (20-54.99% MSS); High (55-74.99% MSS); Very high (> 75% MSS).

Nakamura et al. [52]	Compare arbitrary and individualized speed thresholds on sprinting and repeated-sprints in matches	20-m linear sprint	Mean speed over 20-m linear sprint was used as MSS.	Sprint (> 20 km/h)	Sprint (> 90% MSS)

Núñez-Sánchez et al. [82]	Compare arbitrary and individualized speed thresholds on locomotor demands in matches	40-m linear sprint	The MSS obtained in the 40-m linear sprint was used for the individualization process.	Very low intensity running (0-7 km/h); Low intensity running (7-13 km/h); Medium intensity running (13-18 km/h); High intensity running (18-21 km/h)	< 10% MSS; 10-20% MSS; 20-30% MSS; 30-40% MSS; 40-50% MSS; 50-60% MSS; 60-70% MSS; 70-80% MSS; 80-90% MSS; > 90% MSS.

O’Connor et al. [56]	Compare arbitrary and individualized speed thresholds regarding the impact on non-contact injury	Peak speed in training/match	Peak speed in training/match was considered as MSS for individualization process.	Sprint threshold (> 24.9 km/h)	Sprint threshold (> 75% MSS; > 80% MSS; > 85% MSS; > 90% MSS; > 95% MSS)

Ortega-Gálvez et al. [42]	Compare arbitrary and individualized speed thresholds on locomotor demands in matches	40-m linear sprint starting and 30-15 Intermittent Fitness test for MAS	The final velocity achieved in the 30-15 Intermittent Fitness test was considered as MAS for individualization. The peak speed attained in the best 10-m split was considered as MSS.	Moderate (13.1-18.6 km/h); Sprint (20 km/h)	Moderate intensity (68% MAS); High intensity (> 87% MAS); Sprint (80% MSS)

Park et al. [24]	Compare arbitrary and individualized speed thresholds on locomotor demands in matches	Spectral Clustering, k-means and Gaussian mixture model	The algorithms were used to identify velocity zones in each computed half match, while linear-mixed modelling determined generic squad thresholds	High-velocity running (Generic: 4 m/s, Bradley and Vescovi: 3.34 m/s); Very-high velocity running (Generic: 5.5 m/s, Bradley and Vescovi: 4.45 m/s); Sprinting (Generic: 7 m/s, Bradley and Vescovi: 5.56 m/s)	k-means: High-velocity running (1.05 m/s); Very-high velocity running (2.10 m/s); Sprinting (3.60 m/s); Gaussian mixture model: High-velocity running (0.56 m/s); Very-high velocity running (1.53 m/s); Sprinting (3.05 m/s); Spectral Clustering (*β* = 0.1): High-velocity running (3.46 m/s); Very-high velocity running (5.29 m/s); Sprinting (6.26 m/s); Spectral Clustering (*β* = 0.01): High-velocity running (3.54 m/s); Very-high velocity running (5.38 m/s); Sprinting (6.30 m/s); Spectral Clustering (*β* = 0.001): High-velocity running (3.56 m/s); Very-high velocity running (5.39 m/s); Sprinting (6.30 m/s); Spectral Clustering (*β* = 0): High-velocity running (3.58 m/s); Very-high velocity running (5.41 m/s); Sprinting (6.27 m/s);

Rago et al. [46]	Analyze the relationship between locomotor and physiological demands, while considering arbitrary and individualized speed thresholds in matches.	Yo-Yo intermittent recovery test level 1 and peak speed attained in training sessions.	The final velocity attained in the Yo-Yo intermittent recovery test level 1 was considered for the estimation of the MAS. The peak speed attained in training sessions was considered as the MSS. The ASR was calculated from the difference of MSS and MAS.	Moderate speed running (14.4-19.8 km/h); high-speed running (19.9-25.1 km/h); sprinting (> 25.2 km/h).	Moderate speed running (80-99.9% MAS); high-speed running (100%MAS-29%ASR); sprinting (> 30% ASR).

Rago et al. [14]	Compare arbitrary and individualized speed thresholds on locomotor demands in matches	Yo-Yo intermittent recovery test level 1 and peak speed attained in training sessions.	The final velocity attained in the Yo-Yo intermittent recovery test level 1 was considered for the estimation of the MAS. The peak speed attained in training sessions was considered as the MSS. The ASR was calculated from the difference of MSS and MAS.	Moderate speed running (14.4-19.8 km/h); high-speed running (19.9-25.1 km/h); sprinting (> 25.2 km/h).	Moderate speed running (80-99.9% MAS); high-speed running (100%MAS-29%ASR); sprinting (> 30% ASR).

Reardon et al. [16]	Compare arbitrary and individualized speed thresholds on locomotor demands in matches	Peak speed attained in matches.	The peak speed attained in matches was considered as the MSS.	High speed running (5 m/s^2^).	High speed running (5 m/s^2^ divided by the average of MSS of the group).

Scanlan et al. [83]	Compare arbitrary and individualized PlayerLoad thresholds in training sessions.	Peak instantaneous PlayerLoad intensity recorded in training.	The peak instantaneous PlayerLoad intensity was used to individualized threshold.	PlayerLoad zone 1 (0-1 A.U.); PlayerLoad zone 2 (1-2 A.U.); PlayerLoad zone 3 (2-3 A.U.); PlayerLoad zone 4 (3-4 A.U.); PlayerLoad zone 5 (4-6 A.U.); PlayerLoad zone 6 (6-10 A.U.).	PlayerLoad zone 1 (0-10% peak PlayerLoad); PlayerLoad zone 2 (10-20% peak PlayerLoad); PlayerLoad zone 3 (20-30% peak PlayerLoad); PlayerLoad zone 4 (30-40% peak PlayerLoad); PlayerLoad zone 5 (40-60% peak PlayerLoad); PlayerLoad zone 6 (60-100% peak PlayerLoad).

Scott et al. [17]	Determine dose-response relationship between locomotor and physiological demands while use arbitrary and individualized speed thresholds in training sessions.	40-m linear sprint, the modified version of Montreal Track Test (VAM-EVAL) and the Yo-Yo Intermittent Recovery Test level 1	The best peak speed obtained in the splits of 10-m were considered as MSS. The last stage attained at VAM-EVAL and the Yo-Yo Intermittent Recovery Test level 1 were used to estimate the MAS.	High-speed running (12.67 km/h); Very high-speed running (17.82 km/h)	High-speed running (80%MAS at VAM-EVAL; or 80% at Yo-Yo Intermittent Recovery Test level 1; or 50% MSS); Very high-speed running (100%MAS at VAM-EVAL; or 100% MAS at Yo-Yo Intermittent Recovery Test level 1; or 65% MSS)

Scott et al. [47]	Examine the dose-response relationship between match-player demands and ratings of fatigue and soreness, while using arbitrary and individualized thresholds.	40-m linear sprint, and the 30-15 Intermittent Fitness Test	The fastest sprint in splits of 10-m was considered as the MSS. The final velocity of 30-15 Intermittent Fitness Test was estimated as a MAS measure. The ASR was calculated based on the difference between MSS and MAS.	High-speed running (12.5 km/h); Very high-speed running (19.0 km/h); Sprinting (22.5 km/h).	High-speed running (60% MAS; 50% MSS); Very high-speed running (80% MAS; 65% MSS); Sprinting (30% ASR; 80% MSS).

Siegle et al. [54]	Analyze inter-individual differences in the locomotor speed and compare with general approach in match.	Q-Q-Plots	Q-Q-Plots were used to graphical inspection of intersection point.	Walking (qualitative approach); Jogging (qualitative approach); Cruising (qualitative approach); Sprinting (qualitative approach).	Walking/Jogging (intersection point in movement velocity, average: 2.06 m/s^2^); Jogging-Cruising/sprinting (intersection point in movement velocity, average: 4.53 m/s^2^).

Taylor et al. [48]	Compare the training load in different age-groups, while using arbitrary and individualized speed thresholds.	30-m linear sprint, and the 30-15 Intermittent Fitness Test	The peak speed at 30-m linear sprint was used as MSS. The final velocity of 30-15 Intermittent Fitness Test was estimated as a MAS measure.	High-speed running (> 19.8 km/h); Sprint running (> 25.2 km/h).	High-speed running (87% MAS); Sprint running (80% MSS).

Taylor et al. [84]	Identify the dose-response relationship between training load measures, while considering arbitrary and individualized speed thresholds.	Incremental treadmill test	The velocities at 2 mmol/L and 4 mmol/L lactate were estimated to individualization of speed thresholds.	High-speed distance (> 15 km/h); Very high-speed distance (> 18 km/h).	High speed distance thresholds (velocity at 4 mmol/L lactate, range: 8.7 to 13.1 km/h)

Taylor et al. [85]	To assess the relationships between external and internal load ratios, while considering arbitrary and individualized speed thresholds.	Incremental treadmill test	The velocities at 2 mmol/L and 4 mmol/L lactate were estimated to individualization of speed thresholds.	High-speed distance (> 15 km/h); Very high-speed distance (> 18 km/h).	High speed distance thresholds (velocity at 4 mmol/L lactate, range: 8.7 to 13.1 km/h)

ASR: anaerobic speed reserve; Aust. Foot.: Australian Football; Comp.: competitive; GPS: Global Positioning System; H: hockey; IU: information unavailable; LPS: local positioning system; M: men; MAS: maximal aerobic speed; MSS: maximal sprint speed; N: number of participants; RCT: Respiratory compensation threshold; UK: United Kingdom; USA: United States of America; W: women.

The term “high intensity running” was defined differently across the studies. Namely, it was classified as the second ventilatory threshold [26] [38], the between lactate threshold-80%MSS [39] [40], the between RCT and MAS [41], > 87% MAS [42], 100% MAS, 30% ASR and 20 to 35 W/kg metabolic power zone [43]. The term “high speed running” was defined as meters covered above MAS [11] [44], (50–70% MSS) [45], RCT-95% of maximal oxygen uptake, 80–99% MAS, 50–59% MSS 80–99% MAS [19], 100%MAS-29%ASR [46] [14], 5 m/s^2^ divided by the average of MSS of the group [16], 80%MAS at VAM-EVAL, 80% at Yo-Yo Intermittent Recovery Test level 1, 50% MSS [17], 60% MAS, 50% MSS [47] or 87% MAS [48]. The term “high intensity accelerations” were consistently classified as > 75% maximal acceleration [49] [50] [51]. The term “sprint” was classified as > 90% MSS [52] or 80% MSS [42] [48].

**FIG. 4:**
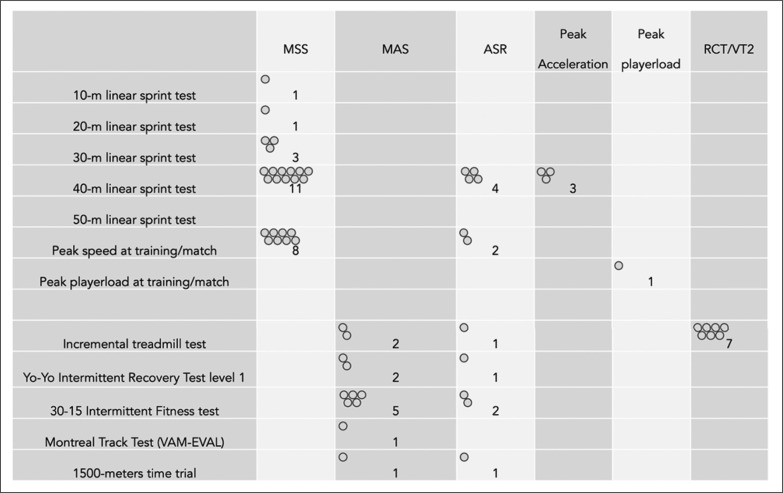
Interaction between physical fitness assessment and main outcomes used in the individualized running speed thresholds. MSS: maximal sprint speed; MAS: maximal aerobic speed; ASR: anaerobic speed reserve; RCT: Respiratory compensation threshold; VT2: second ventilatory threshold. Open circles represents the number of studies.

Figure 4 represents the list of tests used for assessing the measures that were used for establishing the standardized running speed thresholds. The 40-m linear sprint was the test most commonly used to assess MSS (n = 11), followed by the peak speed attained by players in training sessions/matches (n = 8). Moreover, the 40-m linear sprint test was the only test used for assessing peak acceleration (n = 3).

**FIG. 5:**
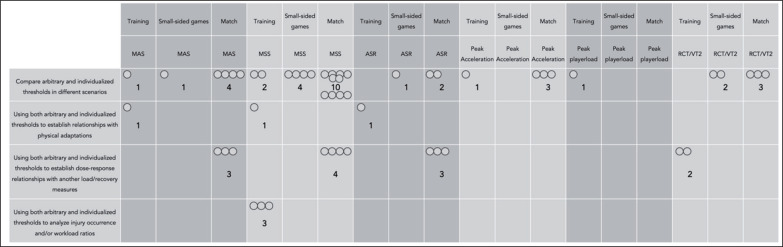
Evidence gap map regarding main topics of study and main measures used for establishing the individualized running speed threshold. MSS: maximal sprint speed; MAS: maximal aerobic speed; ASR: anaerobic speed reserve; RCT: Respiratory compensation threshold; VT2: second ventilatory threshold. Open circles represents the number of studies.

Figure 5 presents the EGM of individualized running speed thresholds compared with arbitrary thresholds. Of the 36 included articles, 27 (75%) centered on the use of arbitrary and individualized running speed thresholds to describe the locomotor demands on players, while 5 (13.9%) centered on establishing relationships between arbitrary and individualized running speed thresholds and training load measures. Three articles (8.3%) used the approaches to establish relationships with injury likelihood or occurrences, and just one study (2.8%) focused on establishing relationships between arbitrary and individualized running speed thresholds and physical fitness adaptations.

## DISCUSSION

Most individualized approaches to establishing running speed thresholds in team sports are focused on the measuring of physical fitness to meet the locomotor demands of those sports. Among other measures, MSS, MAS and respiratory compensation threshold were the main measures used for individualization of running speed thresholds. One of the notable trends found amongst the articles was the diversity of individualized approaches used, with few studies focusing on determining the best method of individualization or comparing different methods to define which might be the most appropriate. Hereafter, the discussion will center on the methodological characteristics and potential bias found during the review.

### Participation characteristics

There was a lack of consensus on the use of terms (e.g., elite, professional) to describe study participants amongst the included studies. Such terms help to provide relevant information about the competitive level of study participants, however, populations such as youth athletes are not easy to classify given their relative lack of experience and variation between countries and sports. This may also be applicable at the so-called “elite” level as professional status can relate to having competed in any one of several different tiers of varying playing standard [53]. In an effort to improve the standardization of athletes’ competitive levels, we recommend that researchers follow the Participant Classification Framework [33] which categorizes players based on their level of practice, volume of training and ranking. The organization of study participants into well-defined tiers may help to standardize information for the scientific community, leading to more accurate appraisals of studies such as those in the current scoping review and the subsequent development of more useful knowledge that can be transferred to practitioners. In this scoping review, we have attempted to classify study participants based on the aforementioned framework, however, in some cases, this was challenging because basic information, such as training and match frequency and hours of training per week, were not available in the gathered studies. More accurate information such as participant skill level or level of competition (tier of league structure, competitions in national or international play) are highly important to better characterize samples. Most of the studies in this review were conducted in tiers 2 (trained/developmental) and 3 (highly trained/national level) indicating that it remains difficult to undertake observational studies in international level or world class athletes. More research should focus on the elite level of sport with studies on world-class female soccer players [24] and the finalists of the men’s world cup [54] being excellent examples of this.

In relation to the diversity of origins of the studies in this review, no studies came from Africa or Asia and only a small number came from the Americas. Of the 36 included studies, 25 were from continental Europe and six came from Australia, indicating that research on this topic is concentrated in specific regions of the world. This may constitute a potential issue for the generalizability of research results and may compromise the establishment of similar performance benchmarks in other regions of the world. Another significant imbalance in the gathered data related to the diversity of team sports that were studied. Soccer was the most commonly studied (n = 25) and was followed by rugby (n = 5) in a distant second place. This makes it difficult to consolidate findings for sports other than soccer as the amount of analyzable evidence is small. In addition to this, another limitation is the sample size in published studies. The number of participants per study ranged from eight to 90. There was a notable absence of the rationale for the various sample sizes utilized in the studies. Even considering the difficulty of performing observational studies in competitive sporting environments, it must be acknowledged that small sample sizes and highly specific contexts can compromise the generalizability of evidence extracted from the analyses. In addition to this, it is highly important that researchers report effect sizes to add context to the outcomes of their hypothesis testing [55]. Finally, another reporting issue which must be improved upon relates to the sampling strategies utilized by researchers. Most investigations comprise of convenience samples with data from just a single team usually analyzed thus undermining the generalizability of this data. Studies with larger samples that are obtained via more robust sampling strategies should be prioritized by researchers. However, another challenge that may emerge in such scenarios is the replicability of the conditions in any given scenario (which, albeit, may be relatively simple to guarantee if the sample comprises of just one team).

Although several studies reported the characteristics of the participants such as body mass and height, most of the included articles did not report this information clearly making it difficult to fully evaluate the extant evidence and compare results for future research. Likewise, although the requirement to protect sensitive or personal data is understandable, it is vital to indicate the sex of study participants as physical fitness and running speed thresholds may vary based on this characteristic [12]. This is also vital in terms of up-holding the replicability of a given study. From the minority of studies which reported the sex of participants, men were more researched (n = 12) than women (n = 6).

### Sample collected

A highly diversity body of data were collected from the included studies. As an example, training volume varied from a minimum of two sessions [11] to a maximum of 114 [56]. Similarly, the number of matches analyzed varied from one (single match) [54] to 52 (collected over three years) [24]. Aside from the substantial difference in the amount of data collected, other issues were also apparent. For example, the period of observation was unreported in six studies with the remaining typically failing to provide any additional contextualization such as the period of the season the data was collected in and the schedule of matches that the teams were exposed to. In future studies, we recommend that researchers accurately describe the period of observation with relevant dates and information on the specific period and the content of the training week(s). Such information can be added as a supplementary file to journal submissions. Encouragingly, all the gathered studies reported on the brand and model of the instruments used to collect data with the accuracy and level of measurement precision also very well described.

### Physical fitness assessment – context-related information

Most (34/36) of the included studies used physical fitness assessment or performance analysis elements to individualize speed running thresholds. However, a particularly vital methodological issue related to the lack of accuracy in reporting the regularity of these assessments. Most of the studies used more than a single game to analyze running speed thresholds, and the range of the observation periods varied from two weeks [39] to three years [24]. Information about the regularity of the assessments and, most particularly, the time between the assessments and the range of matches analyzed was surprisingly scarce in most of the articles. Some studies reported the exact time of assessment (e.g., start of the pre-season or the week before the matches being assessed) [44, 52] and others detailed the regularity of assessment (e.g., six measurements in six weeks) [41, 19]. The availability of more accurate timelines, such as figures or supplementary files with schedules, could make it easier to identify which matches were associated with each fitness assessment. For example, in the case of an assessment performed on, say, 30^th^ October, it could be questioned as to whether that assessment would be relevant to the six subsequent scheduled matches or to the three matches before and the three matches after 30^th^ October. This would be particularly relevant during periods of the season when congestion in the match schedule results in multiple games being played within a very short timeframe [57]. Questions such as these arise from studies’ reporting processes which can compromise the replicability of the methods.

Another issue commonly considered to cause a risk of bias is the absence of information on players’ personal habits at the time of the assessment. Studies tended not to report many important factors related to readiness and performance strategies such as players’ sleep habits (i.e., number of hours and quality of sleep the night before assessment) [58], the composition of dietary intake [59], recovery status (i.e., rest times, ratings of perceived exertion) [60] or the sequence of how the tests were conducted. Moreover, it is also important to emphasize the need to report the validity and reliability of the physical fitness tests employed in each study [61].

### Physical tests – specificities and methodological considerations

In the gathered studies, it was apparent that the individualization process was fundamentally associated with the estimation of MSS or the analysis of a cardiorespiratory marker. The 40-m linear sprint was the most utilized test, featuring in 11 studies and this was followed by the 30-m linear sprint. The 40-m linear sprint appears to be an appropriate distance with which to identify MSS regardless of the sport in question [13, 62]. Moreover, the validity and reliability of linear sprint tests are also very high [62].

Despite the above, some methodological issues were found during the inspection of the articles. For example, when measuring sprint speed, some researchers have placed photocells every 10-m [19] or 20-m [52] along the plotted course to estimate the average running speed of a participant over the chosen distance. However, previous research has revealed that photocells that are positioned every 5-m appear to be a more accurate way to estimate average running speed in comparison to the gold-standard radar gun [63]. It is nonetheless worth noting that measuring average speed in splits of 10-m or 20-m may underestimate the peak speed which a performer achieves and this can result in the inaccurate individualization of running speed thresholds. Moreover, it was commonly observed that there was an absence of detail regarding the method for setting (e.g., split, parallel), the height of photocells as well as determining the distance between the starting line and the first pair of photocells. These factors can also be confounding variables since the starting position of the participant can affect the final recorded time in a sprint test [64]. So too can the height of photocells [65] and the distance between the foot and the first pair of photocells and we encourage researchers to control for and report these factors when conducting studies.

Although the use of a radar gun could be the most recommended approach, the major alternative method to using photocells in the included studies was GPS. Previous studies suggest that GPS with 10 Hz (the most widely used in the gathered articles) can provide valid and reliable information about a player’s peak speed [66]; however, GPS can present some fluctuations in reliability level depending on the position of the device [67]. Accordingly, it is important to detail how a given GPS was used and, in the report, to highlight the accuracy and precision level for estimating peak speed.

A further question associated with the individualization process was the test used to estimate cardiorespiratory fitness. MAS is a marker that was used in eleven of the included articles. However, MAS was estimated using a variety of different measures and instruments such an incremental treadmill test, the Yo-Yo Intermittent Recovery test, the 30–15 Intermittent Fitness Test, the Montreal track test, and the 1500-meter time trial. This can introduce substantial heterogeneity between studies as output for this marker is particularly protocol-dependent (e.g., variations in time of exposure per velocity, transition rhythm) meaning that the variability of the outcome can drastically change the final interpretation and individualization of thresholds [20]. Indeed, a field-based test such as the 30–15 Intermittent Fitness test can overestimate MAS and the overall effect of repeated changes of direction in the test can affect the final score [68].

### Definition of the thresholds based on physical fitness measures

Multiple different approaches to establish the individualized running speed thresholds were observed in the gathered studies. The most common were MSS, MAS and the respiratory compensation threshold. Additionally, ASR, maximal acceleration, and maximal player load were also used. As an alternative, Q-Q plots or machine learning algorithms were utilized. The methods mentioned above may extensively impact upon the variables in a typical match analysis such as high-intensity running distance, high-speed running distance, and sprinting distance.

The lack of definition in the approach to speed threshold individualization was apparent in the gathered studies. As an example, sprinting was classified as both > 90% [52] and > 80% [42, 48] of MSS as estimated in a test, or in the peak speed observed during a training session or match. Currently it is unclear which of these standards constitutes a sprint action. One advantageous reason for using MSS is the lower level of variability that can be observed across time [69]. This stability gives the measure a level of consistency that may not be possible when using MAS.

The use of MAS for establishing the threshold of high-speed running was observed in different studies. For some authors, high-speed running was the point at which MAS was attained [11, 44], while others defined this as being between 50–70% [45] or 80–99% of MAS [19]. A recent systematic review [70] adequately characterizes this methodological dilemma. Furthermore, although the same locomotor demands can be placed on two individuals, the associated physiological demands of movement can vary, thus implying a different physiological representation of high-speed running. Moreover, since MAS is protocol-dependent, this process becomes even more complex to address. Finally, physical qualities can vary in the short term (based on the applied stimulus) [71, 72] meaning that if some forms of physical fitness are not tested every six weeks, it can be challenging to set thresholds that are aligned to a player’s capabilities at the time of match data capture and analysis. This also raises the issue of ‘training residuals’ and the effect that they might have on the estimation of running speed thresholds. Whilst it has been demonstrated that physical qualities such as aerobic endurance and maximal strength can remain for up to 30 days following the reduction or cessation of training, the residual of maximal speed training, and its associated physiological adaptations, is only five days in duration [23]. This means that in order to ensure that a player’s running speed threshold remains as accurate as possible throughout a season, some form of maximal sprint stimulus must occur at least every five days to retain maximal performance of this particular physical quality. If this does not occur, theoretically, a player could lose sprinting speed and subsequent estimations may be inaccurate relative to their best potential performance.

### Future research

In line with the article of Beato et al [13], the commentary of Drust [73] and the letter to the editor of Kavanagh and Carling [74], more effort is required to consolidate methodological approaches to the study of individualized running speed thresholds in team sports. There are clear methodological concerns in the published literature on this topic which we discuss in the current scoping review. Accordingly, some recommendations should be considered by researchers who wish to progress work in this area. In relation to study participants, it is important to increase sample sizes, create more representation across competitive levels and standardize reporting on competitive level, training frequency, player origin and context of practice. Considering measurement instruments, it is important to detail accuracy and precision ensuring that reliability and validity are at the forefront of researchers’ effort to characterize running speed thresholds. Regarding the reporting of study details, it is important to provide a timeline that indicates the exact point of observation using dates and the specific juncture at which data collection occurred. To do this, researchers might perhaps consider adding supplementary files to their journal submissions. Also, it is important to contextualize the data collection process, namely controlling confounding variables of physical fitness assessments. To do this, researchers could use surveys of sleep, standardize players’ nutritional intake, control the effects of environmental conditions and measure the readiness of their participants to perform. The regularity of fitness assessments must also be improved in terms of the amount of elapsed time between measurements.

Future research must focus on comparing different individualization approaches (e.g., using multiple measures combined or isolated), testing across different periods of time (between the assessment and utilization) and identifying the practical effects on workload and injury risk. Moreover, clarification of the debate on the use of physical fitness markers versus machine learning that uses standards based on players’ match demands can also be focused on further. Additionally, analysis of the impact of moderators and mediators such as time of the season, type of population and players’ level of training experience must be also considered.

### Limitations of the scoping review

The current scoping review may present some limitations that should be highlighted. One of these limitations relates to the utilized search strategy and eligibility criteria. We executed a search strategy that our group of authors and experts unanimously accepted. However, as with any other search strategy, this may not uncover all eligible articles. Even so, we have used a comprehensive search strategy to mitigate this potential source of bias. Despite the use of two independent researchers to select the articles, in addition to world experts whose status is based on measures of overall scientific impact, this may not ensure that all relevant articles were identified. We focused on scientific studies that included both individualized and non-individualized running speed thresholds which meant that some articles, which focused only on individualized running-speed, were not included in the review. However, our methodological approach was designed to conform to all aspects of the PRISMA statement which represents a progressive methodological step in relation to the execution of a traditional scoping review. Finally, we established a rationale for the presentation of our results which consisted of exploring the methodological approaches made by the original studies, and not explicitly focusing on the primary outcomes reported in these studies.

## CONCLUSIONS

The current scoping review summarizes studies that reported on both arbitrary (absolute) and individualized running speed thresholds in team sports. Of the included studies, most used arbitrary and individualized running speed thresholds to compare distances covered in match or training sessions. However, there were also some studies that used individualized running speed thresholds to determine the dose-response relationship between locomotor demands and physiological responses, or to associate the exposure of locomotor demands with injury occurrence. In terms of methodological issues, most of the studies used physical fitness or physical performance measures to individualize running speed thresholds. However, the main risk of bias identified in terms of methodological quality was the regularity of assessment and its possible impact on the measurement. Additionally, information about the context of assessments and their replicability should be improved. This specifically relates to authors’ providing information on player readiness, well-being and physical fitness status as well as the reliability of the associated data. Moreover, the techniques used to individualize were not consistent between studies and there was a specifically wide diversity of outcomes and tests used to ascertain running speed. In many cases, the established thresholds differed even when measured with the same tool or instrument (i.e. MAS). The most common measures used to individualize running speed thresholds were MAS, MSS, and the respiratory compensation threshold. Future research is needed on methodological issues and biases related to data collection and to define the most appropriate way to individualize running speed thresholds.

## Supplementary Material

Arbitrary absolute vs. individualized running speed thresholds in team sports: A scoping review with evidence gap map
