# Supplementary material for: Arbitrary absolute vs. individualized running speed thresholds in team sports: A scoping review with evidence gap map
Source: Biol Sport. 2023 Feb 1;40(3):919–43. doi: 10.5114/biolsport.2023.122480 (PMC10286616; doi:10.5114/biolsport.2023.122480)
Supplement: Arbitrary absolute vs. individualized running speed thresholds in team sports: A scoping review with evidence gap map [file JBS-40-122480-s1.pdf]

SUPPLEMENTARY MATERIAL 1.

LIST OF INCLUDED AND EXCLUDED ARTICLES IN THE FULL-TEXT ROUND OF SELECTION.

1	2	3	4	5	6	7
**Title**	**(i) population: team sports players from any age and from both sexes integrated into team routines (i.e., not injured or with any reported pathology or health problems; also not included disabled or adapted sports);**	**(ii) exposure: exposed to analysis of individualized running speed thresholds in training sessions and/or matches;**	**(iii) comparator: exposed to traditional arbitrary running speed thresholds in training sessions and/or matches;**	**(iv) Outcome(s): the time and/or distance and/or percentage of time and/or distance spent in different running speed thresholds (either in arbitrary/fixed or individualized thresholds);**	**(v) study design: observational studies or interventions (both single-arm [if with two different metrics, for example, individualized vs. arbitrary] and multi-arm investigations)**	**Included**
Application of individualized speed thresholds to interpret position specific running demands in elite professional rugby union: A GPS Study	Yes	Yes	Yes	Yes	Yes	Yes
Match analysis of U9 and U10 english premier league academy soccer players using a global positioning system: relevance for talent identification and development	Yes	No	No	No	Yes	No
Individualisation of time-motion analysis: A method comparison and case report series	Yes	Yes	Yes	Yes	Yes	Yes
Use of Individual Relative Thresholds to Assess Acceleration in Young Soccer Players According to Initial Speed	Yes	Yes	Yes	Yes	Yes	Yes
Comparison of player-dependent and independent high-speed running thresholds to model injury risk in football	Yes	Yes	Yes	Yes	Yes	Yes
Individualizing acceleration in english premier league academy soccer players	Yes	Yes	Yes	Yes	Yes	Yes
Relationship between external load and perceptual responses to training in professional football: Effects of quantification method	Yes	Yes	Yes	Yes	Yes	Yes
High-intensity acceleration in soccer. Why is the evaluation method important?	Yes	Yes	Yes	Yes	Yes	Yes

SUPPLEMENTARY MATERIAL 1.
Continue.

1	2	3	4	5	6	7
Measurement properties of external training load variables during standardised games in soccer: Implications for training and monitoring strategies	Yes	No	No	No	Yes	No
The relation between movement velocity and movement pattern in elite soccer	Yes	Yes	Yes	Yes	Yes	Yes
Individualized and fixed thresholds to demarcate playerload intensity zones produce different outcomes	Yes	Yes	Yes	Yes	Yes	Yes
Greater association of relative thresholds than absolute thresholds with noncontact lower-body injury in professional australian rules footballers: Implications for sprint monitoring	Yes	Yes	Yes	Yes	Yes	Yes
Use of Relative Speed Zones Increases the High-Speed Running Performed in Team Sport Match Play	Yes	Yes	Yes	Yes	Yes	Yes
Match-to-match variability in high-speed running activity in a professional soccer team	Yes	Yes	Yes	Yes	Yes	Yes
Dose-response relationship between external load and wellness in elite women's soccer matches: Do customized velocity thresholds add value?	Yes	Yes	Yes	Yes	Yes	Yes
Default and individual comparison of physiological responses and time-motion analysis in male and female soccer players during small-sided games	Yes	Yes	Yes	Yes	Yes	Yes
The use of individualized speed and intensity thresholds for determining the distance run at high-intensity in professional soccer	Yes	Yes	Yes	Yes	Yes	Yes
Local Positioning System-Derived External Load of Female and Male Varsity Ice Hockey Players During Regular Season Games	Yes	Yes	Yes	Yes	Yes	Yes

SUPPLEMENTARY MATERIAL 1.
Continue.

1	2	3	4	5	6	7
An individual approach to monitoring locomotive training load in English Premier League academy soccer players	No	No	No	No	No	No
The dose-response relationship between training load and aerobic fitness in academy rugby union players	Yes	Yes	Yes	Yes	Yes	Yes
Individualization of Time-Motion Analysis: A Case-Cohort Example	Yes	Yes	Yes	Yes	Yes	Yes
Individualized speed threshold to analyze the game running demands in soccer players using GPS technology	Yes	Yes	Yes	Yes	Yes	Yes
External Training Demands in Women's Varsity Rugby Union Players Quantified by Wearable Microtechnology With Individualized Speed Thresholds	Yes	Yes	No	Yes	Yes	No
The use of generic and individual speed thresholds for assessing the competitive demands of field hockey	Yes	Yes	Yes	Yes	Yes	Yes
Physiologically based GPS speed zones for evaluating running demands in Women's Rugby Sevens	Yes	Yes	Yes	Yes	Yes	Yes
Individualisation of speed thresholds does not enhance the dose-response determination in football training	Yes	Yes	Yes	Yes	Yes	Yes
Monitoring Matches and Small-sided Games in Elite Young Soccer Players	Yes	Yes	Yes	Yes	Yes	Yes
The challenge of evaluating the intensity of short actions in soccer: A new methodological approach using percentage acceleration	Yes	Yes	No	Yes	Yes	No
Repeated-Sprint Sequences during Female Soccer Matches Using Fixed and Individual Speed Thresholds	Yes	Yes	Yes	Yes	Yes	Yes
Influence of the number of players and the relative pitch area per player on heart rate and physical demands in youth soccer	Yes	Yes	Yes	Yes	Yes	Yes

SUPPLEMENTARY MATERIAL 1.
Continue.

1	2	3	4	5	6	7
Number of Players and Relative Pitch Area per Player: Comparing Their Influence on Heart Rate and Physical Demands in Under-12 and Under-13 Football Players	Yes	Yes	Yes	Yes	Yes	Yes
Individual vs general time-motion analysis and physiological response in 4 vs 4 and 5 vs 5 small-sided soccer games	Yes	Yes	Yes	Yes	Yes	Yes
Dose-Response Relationship Between Training Load and Changes in Aerobic Fitness in Professional Youth Soccer Players	Yes	Yes	Yes	Yes	Yes	Yes
Reliability and validity of integrated external and internal load ratios as measures of fitness in academy rugby union players	Yes	Yes	Yes	Yes	Yes	Yes
The use of relative speed zones in Australian Football: Are we really measuring what we think we are?	Yes	Yes	Yes	Yes	Yes	Yes
Application of Individualized Speed Zones to Quantify External Training Load in Professional Soccer	Yes	Yes	Yes	Yes	Yes	Yes
Mind the "Gap": A Comparison of the Weekly Training Loads of English Premier League Academy Soccer Players in Under 23, Under 18 and Under 16 Age-Groups	Yes	Yes	Yes	Yes	Yes	Yes
Motion analysis of U11 to U16 elite English Premier League Academy players	Yes	Yes	Yes	Yes	Yes	Yes
Quantification of the running demands in women`s field hockey using individualized thresholds	Yes	Yes	Yes	Yes	Yes	Yes
Velocity zone classification in elite women's football: where do we draw the lines?	Yes	Yes	Yes	Yes	Yes	Yes
Workload Monitoring in Top-level Soccer Players During Congested Fixture Periods	Yes	Yes	Yes	Yes	Yes	Yes
